# 

**Published:** 1881-03

**Authors:** 


### INDEX TO VOLUME XVIII.

PAGE
Academy of Medicine.......... 154
American Medical Association......... 176
Autiseptic Surgery........... 238
Arsenic in Chorea............... 252
Addison's Disease............... 304
Arsenic, an Antidote for....... 318
Albumen in the Urine......... 318
American Gynæcological Association.......... 333
Alkaloids, tests of............... 445
Academy of Medicine.......... 479
Arsenic, test for................ 576
Academy of Medicine......... 604
Acid Medicines, caution of.... 63
Anæsthetic, new................. 76
Atlanta Hospital.................. 630
Action of Remedies, a Thesis. 1
Apothecary and Physician..... 765
Albuminuria, a treatise on..... 767
Antiseptic Dressings............. 770
Asphyxiated Infants, restoration of......... 772
Academy of Medicine and Surgery of Kentucky............. 668
Anæsthesia in Labor............ 680
Amblyopia from the Abuse of Tobacco and Alcohol......... 682
Burns and Scalds, early treatment of......... 34
Bromide of Ethyl................. 188
Baltimore Medical Association 209
Benzoate of Soda in Parasitic Disease......... 253
Bloodless Method of Performing Surgical Operations...... 769
British Medical Association.... 412
Brain, Organ of the Mind...... 445
Bromide of Potassium, action of......... 640
Bacteria......... 705
Bright's Disease, treatment of 706
Cincinnati Medical Society,... 7
Chorea aud Whooping Cough.. 114
Cincinnati Medical Society..... 147
Catarrh of the Bladder.......... 189
Cincinnati Academy ot Medicine.........197, 740
Cutaneous Diseases, Pilocarpine in......... 771

PAGE.
Carbolic Acid...................... 194
Catarrh of the Bladder in old people......... 253
Cincinnati Obstetrical Society 259
Cancer Cure........................ 284
Congenital Amaurosis, spontaneously cured......... 316
Celluloid ............................ 361
Catarrh, Naso-Pharyngeal... 384
Congenital Primosis, wound of 513
Chian Turpentine, and its uses in Cancer......... 542
Close of the Year................ 568
Children, Cure and Culture of 570
Cancer, Trichinosis and Tuberculosis......... 591
Cadaveric Alkaloid............... 631
Cystitis, treatment of............ 639
Chloroform Poisoning. .......... 641
Contraction of the Uterine Orifices in Relation to Dysmenorrhæ and Sterility...... 711
Disease, the Germ Theory of.. 29
Domestic Hygiene................ 187
Detroit Academy of Medicine 267
Diphtheria Poison, Vitality of 317
Dipsomania........................ 761
Diseases of the Skin, Practical Treatise of......... 766
Diseases of the Throat and Nose, a Manual of............. 766
Diphtheria.......................577, 463
Dropsy, a case of................ 541
Diseases of the Heart......... 550
Diphtheria, treatment of....... 571
Editorial Correspondence...... 168
Endocarditis........................ 297
Epsom Salts, Disguising the Taste......... 320
Electricity.......................... 440
Eruptive Fevers, Parallelism of......... 758
Ergot, Best Modes of Giving... 446
Ear Diseases, Diagnosis and Treatment of......... 570
Female Pelvic Organs, Anatomy of......... 384
Functional Nervous Diseases, Treatise on Common Forms of......... 445

PAGE.
Fever, Hay.............................. 576
Formulæ, L'union Medicale... 634
Genital Irritation..................... 223
Gynæcological Practice —Are Dilatation Instruments necessary ?.............................. 313
Gonorrhœa, Boracic Acid injected in.............................. 316
Glycerine Cement..................... 317
Ground Air.............................. 354
Gastric Ulcer and Cancer, Differential Diagnosis of... 638
Intussusception of the Bowels 131
Iodoform in Nasal Catarrh and Treatment of Wounds.. 315
Influence of Certain Localities on Health, Particularly Consumption..................... 493
Infantile Constipation........... 635
Intestinal Worms................. 336
Infection.............................. 704
Homœpathy........................... 186
Hypodermic Quinia................ 255
Hydrophobia, a New Remedy for.............................. 313
Jaundice, an Epidemic of...... 6
Leprosy. Etiology of............. 709
Medico-Legal Society............ 24
Midwifery, Curious Case of..... 38
Medical Gathering in New York.............................. 52
Mind Troubles...................... 61
Malarial Pneumonia.............. 73
Medical Society of Georgia... 120
Meteorological Report for April, 1880........................... 122
Medical Authors................... 123
Malarial Poison..................... 713
Medical Society of the County of New York.................... 744
Materia Medica and Therapeutics of the Skin, a treatise on 767
Metric System, Objections to... 133
Medical Association of Delaware County, Ohio............. 157
Memorial to Congress, from Board of Health................ 173
Meteorological Report for May, 1880......................... 185
Management of Children in Sickness and Health,......... 187
Medico-Chirurgical Society of Montreal........................... 213
Metric System..................... 257
Middle Ear, Suppurative Inflammation of.................... 288
Meteorological Report for October............................ 572

PAGE.
Meteorological Report for June, 1880.............................. 250
Meteorological Report for July, 1880.............................. 312
Morphinism.......................... 342
Meteorological Report for August, 1880......................... 385
Meteorological Report for September, 1880..................... 443
Medicine, System of.............. 444
Maize, Stigmata of................ 446
Malaria................................ 483
Michigan State Board of Health.............................. 514
Minutes of the Biological and Microscopical Academy of Natural Sciences............... 536
Meteorological Report for November. 1880..................... 569
Mark Twain's Recipe for New England Pie...................... 576
Malignant Tumors of the Breast.............................. 609
Meteorological Report for December, 1880..................... 633
Malaria in New England........ 688
Medical Legislation and Ethical Changes...................... 703
Meteorological Report for February, 1881...................... 707
Medical Experts.................... 710
New York Academy of Medicine................10. 104, 140, 526
National Board of Health...... 53
New York Pathological Society 144
New Method of Administering Kousso............................ 192
New York Surgical Society..... 323
New York Society of German Physicians........................ 531
New York Pathological Society 595
Oxytocis in Obstetrics........... 624
Occulsion of the Vagina........ 722
Obsteir c Section of New York Academy of Medicine........ 135
Obstetrical Society, Cincinnati 79
Placenta Prævia...... 50, 128, 614
Puerperal Eclampsia, a case of 65
Piscida Erythrina, Jamaica Dogwood.............................. 255
Paralytic Complication, a case of Calculi, with................ 321
Pregnancy of the Stomach, Hystero-Neurosis of........... 751
Pruritus Vulvæ Relieved by Iodide of Potassium.......... 722
Primosis, a New Procedure for 772
Prolonged Gestation............. 773

PAGE.

Pepsine, Notes on........,...... 377
Pharmacopoæ, New.... .......... 383
Physiology, a New School of... 384
Pneumonia, Typhoid Fever and Anæmia in Bellevue Hospital..................... ....... 449
Pregnancy, Sickness and Vomiting of.............................. 492
Pregnancy During the First Three Months..................... 489
Physiological Antagonism between Medicines, Remedies and Diseases..................... 495
Physicians and Druggists ..... 508
Physiology, Humorous.......... 573
Pilocarpin in Asthma............ 640
Pathological Society of Philadelphia......... ......... ......... 643
Pneumonia, treatment of........ 698
Rolla District Medical Society 220
Remedies, Antagonism of....... 564
Raw Meat, Preserving of....... 712
Quarantine and National Board of Health............... 172
Subacute and Chronic Gout, treatment of...................... 43
St. Louis Medical Society...... 95
Sclerotic Acid—Physiological Action and Therapeutic Value of................. ......... 125
Stone in the Bladder............. 129
Syphilis and Marriage........... 767
Sterility in Women............... 159
Spray in Antiseptic Surgery... 191
Sarcoma of the Thigh, Amputation at Hip-Joint for........ 234
Smallpox, a New Treatment of 254
Shortcomings of the Journal... 311
Snake Bite, a cure for............ 314
Sayre's Treatment for Spinal Curvative .......................... 353
Syphilis, Diagnosis of............ 363
Syphilis from the Father, Transmission of.................. 448

PAGE.

St. Louis Medico-Chirurgical Society......... ......... ........ 465
Suffolk District Medical Society.................................. 578
Surgical Diagnosis, Treatise of 570
Surgical Gynæcology............ 633
Squamous Skin Diseases........ 638
Sore Nose, Liniment for........ 640
St Louis Obstetrical and Gynæcological Society...... 675
Stomach Pump..................... 708
Symmetrical Neuralgia in Diabetes............................ 712
Talipes, treatment of............ 223
Tannerism ......... ................ 310
Tar-Water in Obstetrical and Gynæcological Practice...... 385
Thesis on the Action of Remedies.................................. 725
Typhoid Fever...................... 433
Therapeutics, treatise on........ 444
Uræmia treated by Pilocarpin 251
Uterus after Parturition, management of......... .............. 270
Urine of Typhoid Patients... 575
Ulcerating Surfaces and Abscesses .............................. 637
Urinary Analysis.................. 706
Vanderburgh County Medical Society ........... ................ 91
Visual Phenomenon and its Explanation ...................... 127
Vaginal Hysterotomy..............314
Vulvitis Infantalis................. 315
Vaginal Injections, Hot Water 357
Valedictory Address by H. M. O'Daniel..... .......... ......... 716
Virus, Vaccine...................... 576
Volitional Control over Involuntary Acts, curious case of 639
Valuable Medical Prescriptions......... ............. ......... 706
Yellow Fever, origin of......... 242
What Determines the Sex of Children ............................ 686

